# Transforming a clinical study database into a structured database adapted to artificial intelligence applications

**DOI:** 10.1186/s13244-025-02087-2

**Published:** 2026-02-16

**Authors:** Thibault Sauron, Carole Lazarus, Camille Kurtz, Florence Cloppet, Isabelle Thomassin Naggara, EURAD Study Group, Edouard Poncelet, Aurelie Jalaguier-Coudray, Ingrid Millet, Valerie Juhan, Corinne Balleyguier, Caroline Malhaire, Nicolas Perrot, Marc Bazot, Patrice Taourel, Emile Darai, Laure Fournier

**Affiliations:** 1 LIPADE, Université Paris Cité, Paris, France; 2 Philips Research France, Paris, France; 3 Sorbonne Université, AP-HP, Department of Diagnostic and Interventional Imaging, Hôpital Tenon, Paris, France; 4 Université Paris Cité, AP-HP, Department of Radiology, Hôpital Européen Georges Pompidou, PARCC UMRS 970, INSERM, Paris, France; 5 Service d’Imagerie de la Femme, Centre Hospitalier de Valenciennes, Valenciennes, France; 6 Institut Paoli Calmettes, Marseille, France; 7 Lapeyronie Hospital, University of Montpellier, Montpellier, France; 8 Hôpital de la Timone, Marseille, France; 9 Institut Gustave Roussy, Paris, France; 10 Institut Curie, Paris, France; 11 Centre Pyramides, Paris, France; 12 Service de Radiologie, Hôpital Tenon, Assistance Publique–Hôpitaux de Paris, Sorbonne Université, Paris, France; 13 Service de Gynecologie et Obstetrique et Médecine de la Reproduction, Hôpital Tenon, Assistance Publique–Hôpitaux de Paris, Hôpitaux Univesitaires Est Parisien, Paris, France

**Keywords:** Data curation, Clinical trial, Artificial intelligence, Medical computer vision, MRI

## Abstract

**Objective:**

Medical imaging databases suitable for training machine learning/computer vision algorithms are scarce, limiting the potential for development and generalisation of clinical tools. Clinical trial databases are a source of data, known for their high-quality data and reliable annotations. However, they are not tailored to the needs of machine learning or deep learning models. Our objective was to develop a methodology and tools that enable the curation of these databases specifically for the training or testing of artificial intelligence tools.

**Materials and methods:**

MRIs from the French centres of the EURAD clinical trial (MRI of women with pelvic adnexal lesions) were used to constitute the database. We developed the steps required to curate a clinical trial database: definition of inclusion and exclusion criteria, removal of unnecessary data according to the principle of parsimony, quality control, and harmonisation.

**Results:**

A total of 713 patients were included in our study. The directory structure was simplified, and the number of files and folders decreased by 44% and 95% respectively. Only 62 DICOM fields were considered necessary for artificial intelligence (AI) model applications. Quality control was implemented in repeated cycles of automatic checks, followed by a final manual random inspection. Finally, sequence names were harmonised for easy identification when developing models.

**Conclusion:**

Using a clinical trial database, we propose a methodology to build a database suitable to train or test AI algorithms. This study underlines the need for a more global and systematic framework for the secondary use of health data to develop AI imaging tools for patient care.

**Critical relevance statement:**

We propose and detail a framework and tools to curate a clinical trial database to allow secondary use of the high-quality annotated data generated in clinical trials for the training and testing of artificial intelligence models.

**Key Points:**

- Clinical trial imaging databases are not adapted for AI model development.
- A curation process of MRI databases was developed for machine learning applications.
- We share the open-source tools and methodology developed in this study.

**Graphical Abstract:**

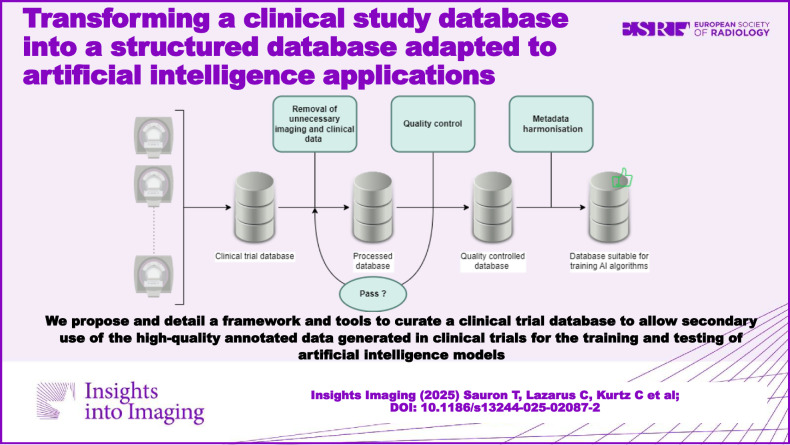

## Introduction

Medical Artificial Intelligence (AI) algorithms can help doctors by improving diagnostic accuracy and automating tasks such as image segmentation, leading to more efficient workflows. In 2023, van Leeuwen et al [1] listed 119 Food and Drug Association (FDA)- and European Commission (EC)-approved AI software applications for radiology. Although deep neural networks are widely used in natural image applications [2–4], their performance in medical settings lags behind. A major obstacle is the lack of large, representative databases. Networks for natural images have been trained on millions of examples [5, 6], while medical databases typically contain only hundreds or thousands of patients, such as the Brain tumor segmentation (BraTs) imaging database [7] or The Cancer Imaging Archive (TCIA) [8], often with limited or lower-quality annotations. Medical data are difficult to collect due to the complexity of annotation, the collection process, and data sensitivity.

Clinical trial databases, however, are commonly compiled both retrospectively and prospectively, maintaining high data and annotation standards. Using them to develop AI algorithms leverages the medical expertise and quality control inherent to clinical trials. Access is restricted to specific frameworks [9], although secondary use is allowed under certain conditions.

For training and validation of machine learning (ML) or computer vision (CV) algorithms, a principle of parsimony should guide database content, collecting only what is strictly necessary. There is increasing concern that AI algorithms may embark identifiable information from training datasets, making it preferable to remove non-essential clinical data and DICOM metadata.

Despite their high standards, clinical trial databases are not directly suited for machine learning (ML) or computer vision (CV) applications. Clinical databases are designed around a primary hypothesis, while ML datasets should be general to support multiple applications. Expanding scope risks reintroducing biases the trial aimed to limit and also challenges the parsimony principle. Furthermore, inconsistencies tolerable to human interpretation can render parts of a machine learning database unusable, making harmonisation and curation critical but challenging steps.

The purpose of this study was to develop and describe a process to transform a clinical trial database into a database that complies with regulatory and technical requirements to allow training of ML/CV algorithms, such as neural networks, which was applied to the EURAD prospective multicenter cohort study [10] as a proof of concept and experimental validation. This clinical trial was designed to assess the accuracy of the ADNEX MR score (now named O-RADS score) for stratifying the risk of malignancy in sonographically indeterminate adnexal masses from MRI images.

## Method for curating clinical trial databases

### Population

**Fig. 1:**
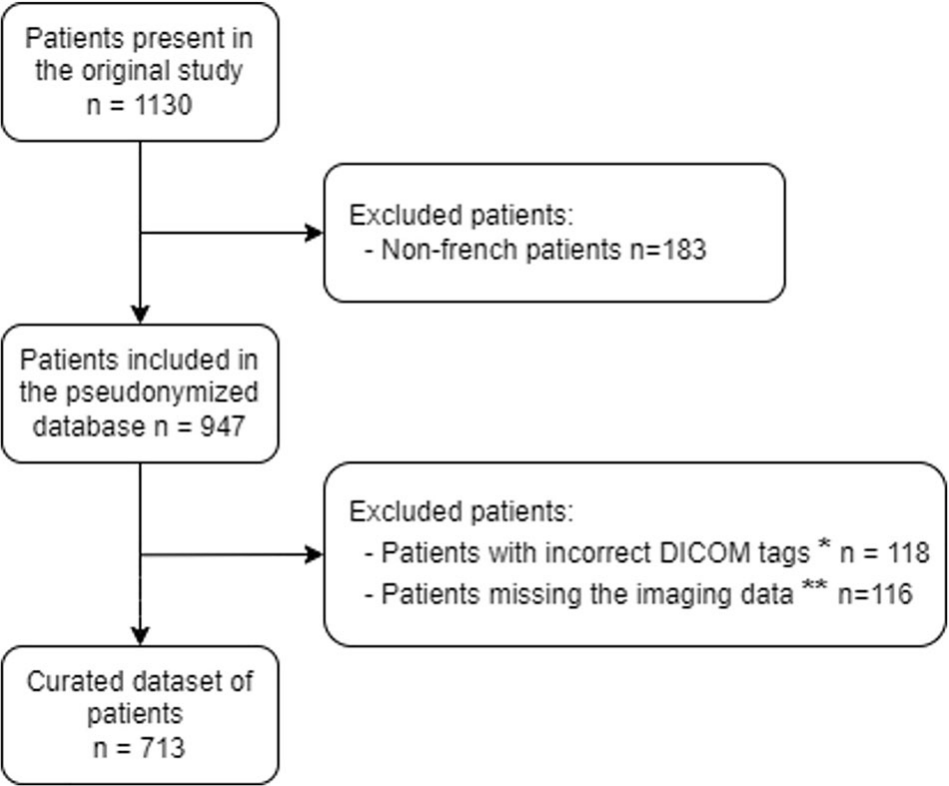
Study population flowchart. *Patients for whom DICOM files were corrupted, unreadable or DICOM tags could not be processed. **Patients for whom none of the 4 required sequences were available: (T2, T1, T1 with fat suppression or T1 post-contrast with fat suppression)

Only French patients from the original EURAD clinical trial database [10] were collected to comply with national regulations. Patients were included if they had at least one out of the following four sequences considered as a minimum requirement: T2-weighted, T1-weighted, T1-weighted with fat suppression and T1-weighted with fat suppression and contrast agent injection. During curation, patients whose DICOM files could not be properly read or curated were secondarily excluded. Patients who had imaging but no clinical data were not excluded, as imaging data on its own can be valuable in some contexts. The flowchart is presented in Fig. 1.

### Curation of the clinical and imaging metadata

The purpose of curation is to “review and validate the annotated data, and resolve any errors or discrepancies” [11]. In our case, some of the curation had already been done during the clinical trial, but steps were needed to make the database compatible with AI applications. The main differences between a clinical trial database and a machine learning-compatible database are shown in Table 1.

**Table 1:** Differences between clinical and machine learning databases

Clinical trial databases	Machine learning databases
Made to be read by doctors	Made to be read by software
Intended to be used in a PACS environment	Only files are provided, final user chooses the environment in which to import them
Uses DICOM format for the largest compatibility	Uses specific format (niftii, nrrd…) for performance
Made to answer one specific question	Made to be as general as possible
Made to be shared with a limited number of known participants	Made to be shared with an unknown number of unidentified participants
Dataset is pseudonymised and can contain identifying data as it is shared with trusted parties	Dataset must be completely anonymised
Complex and inconsistent structure because PACS can harmonise data on the fly	Simple and consistent structure for systematic and automatic reading
High storage space for easy integration in a new environment	Low storage space for easy sharing
Bias reduced from a medical perspective and by reducing scope	Keep all original biases and reduce the ability to control certain biases by removing information

**Fig. 2:**
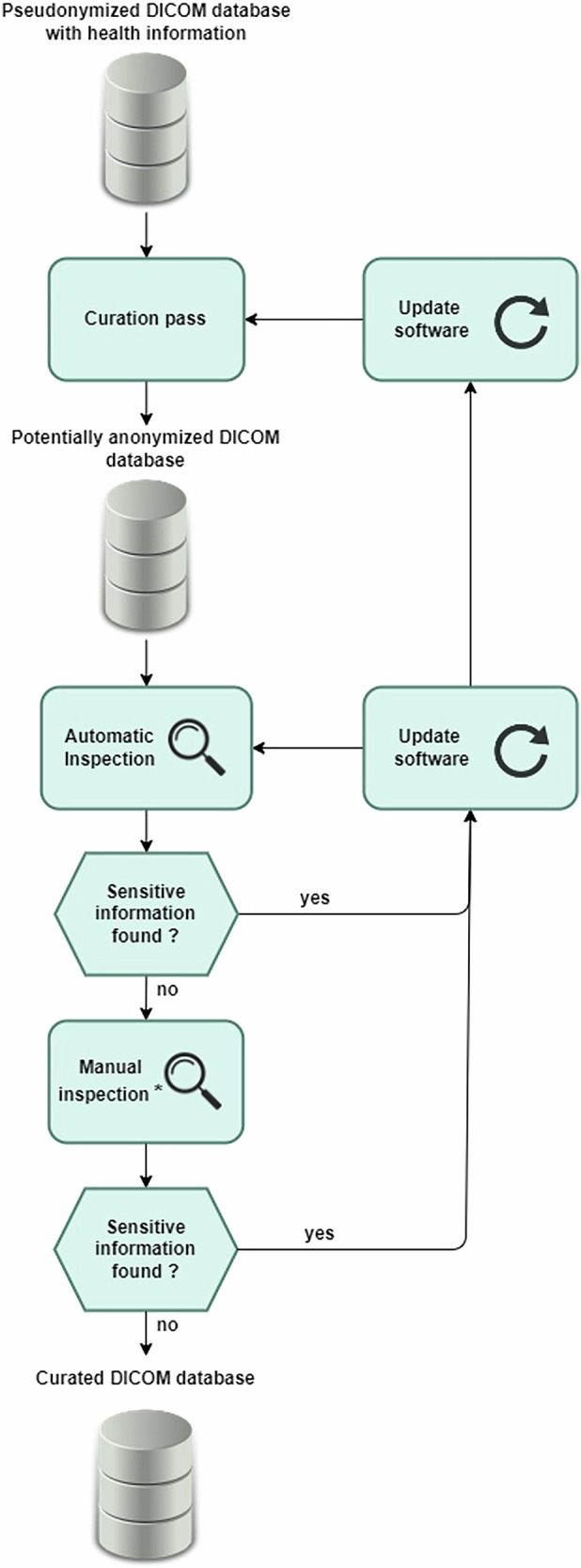
General anonymisation process. Automatic inspection consisted of DICOM tag and sequence name extraction to ensure no unneeded data remained. A manual Inspection was conducted to control the logs for the absence of errors, the list of DICOM tags and of sequence names, on increasingly large numbers of patients and scope as the development progressed. Checks for errors diagnosed during the manual inspection were added to the automatic inspection software to improve the process

First, a senior radiologist with 16 years’ experience in gynaecological imaging (L.F.) defined clinical and imaging data necessary to make the database clinically relevant, while excluding unnecessary data. Second, a curation process was implemented and applied to the EURAD data. This process was divided into three steps, as illustrated in Fig. 2:

- Step 1: Removal of unnecessary imaging and clinical data and assignment of a new study ID;
- Step 2: Quality control to ensure parsimonious curation while retaining relevant information;
- Step 3: Metadata standardisation.

### Step 1: Removal of unnecessary imaging and clinical data

Although the acquired data were already de-identified, all the clinical and imaging metadata were not necessarily relevant for the purpose of training an ML/CV algorithm. As stated above, the principle of parsimony requires that only the strictly necessary data should be retained. Moreover, the greater the number of combined clinical and imaging metadata, the greater the risk of re-identification. Therefore, DICOM tags and clinical data were reviewed by the senior radiologist (L.F.) to identify which fields were needed for future image analysis, while all other fields were deleted or modified. To break the link between the original clinical trial database and the new ML-dedicated database, patients were given a new random ID number, and the correspondence table was destroyed. This strategy was reviewed by the institution’s data protection officer, who determined the data could be considered anonymised, and therefore GDPR did not apply, and no further patient information was required for this study.

#### Patient identifier

In the context of the clinical trial, patient names were pseudonymised with a code of XXXYYY, XXX being an identifier of the institution that emitted the images, and YYY being a numerical identifier of the patient. To sever the link between the patient ID in our database and the original institution, the patient list was shuffled, and patients were assigned a new ID in the form CBIROvXXXX, where CBIR stood for Content-Based Image Retrieval (the final purpose of the database), Ov stood for Ovarian, and XXXX was a random unique number.

#### Clinical data

Among the clinical data, the only fields retained were patient age at the time of the study, size and side of the lesion, histological diagnosis, benign vs malignant class, and imaging signs as interpreted by the local investigator during the clinical trial (presence of cystic portion, presence of tissular portion, T1, T2, DWI signal, ADC value in the tissular portion, perfusion curve types 1–3) [10].

#### DICOM metadata

DICOM fields include mandatory and optional tags and may contain indirectly identifying information. We implemented a strategy whereby we identified tags required for the correct display and quantification of images, as well as some information to control for bias (e.g., manufacturer and year of acquisition). These tags were either left as they were, if considered non-identifying or otherwise transformed. For example, we considered that the date of the imaging study was necessary to control for dataset shifts related to the evolution of acquisition techniques, but only the year was retained to decrease the specificity of the information. All non-required tags were removed. Thus, for every tag, one of three rules was applied:

- “Keep as is” for required tags that were considered non-identifying;
- “Anonymise” for required tags that were potentially at risk of re-identification;
- “Remove” for all other tags.

DICOM files were first analysed, and each field was classified into one of these categories. Anonymisation strategies were designed depending on the nature of the tag. To identify images that might contain directly or indirectly identifying embedded information, the list of sequences available in the database was extracted from the DICOM metadata and manually checked. This step also served to identify and harmonise sequence names. All sequence names that did not correspond to a sequence required for image analysis were deleted during the curation process (e.g., screenshots, time-intensity curves, localisers…). We considered that images covering only the abdomen and pelvis were not inherently identifiable, in contrast to brain images, which require skull stripping to remove identifying features [7, 12].

#### Implementation of the curation script

**Fig. 3:**
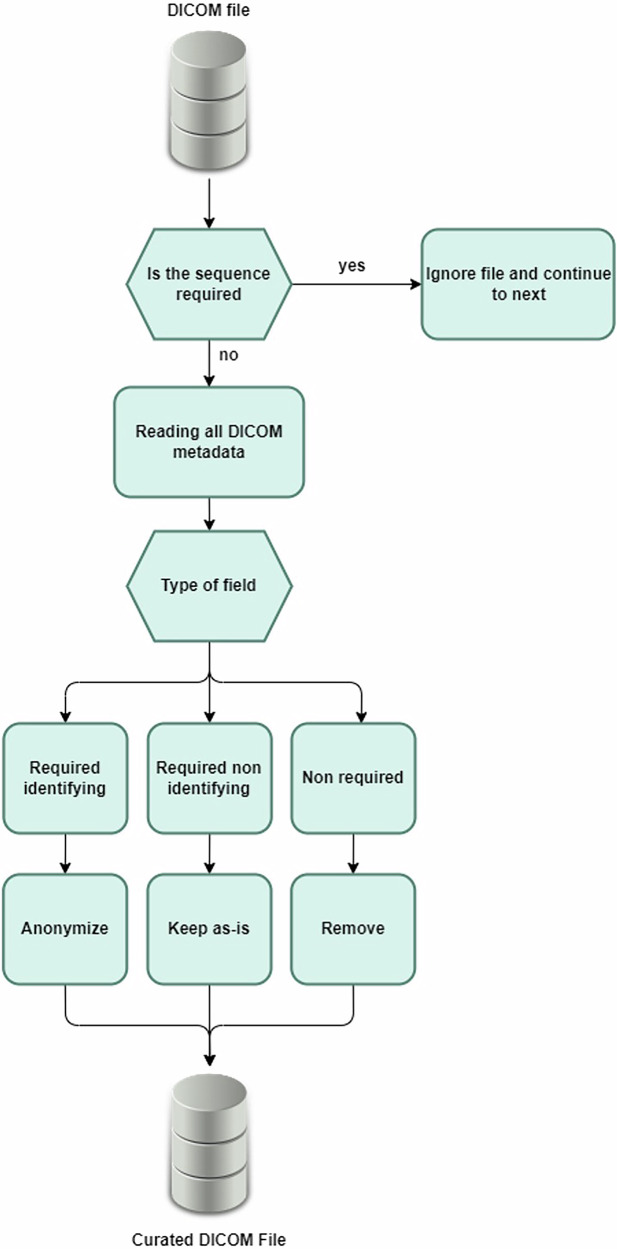
Anonymisation process of a single DICOM file. The file was read, each field was parsed, and actions were taken depending on its type decided by the software user. The file was then written in the destination directory specified by the user

The curation phase was implemented as follows. For each file, all the DICOM tags were read and only the necessary ones were retained, modifying them according to the rules above. All non-essential tags were eliminated, and the resulting modified DICOM file was stored in a separate directory (Fig. 3). Any DICOM file containing sequences not required for analysis was ignored. To ensure traceability, actions taken were logged during the process, including the name of the opened and saved file, the number of DICOM fields before and after curation, and whether an error occurred during the process. These logs were manually reviewed to identify any potential errors. Once this verification was complete, the log file was destroyed to sever the tie between the original file names and the new file names. The curation process was applied to all sub-directories within the database, and all image files were stored directly one level below the patient folder. This step was implemented to ensure that the directory structure itself could not be used as an identifying factor, as it may vary across institutions.

### Step 2: Quality control

Manually checking every field of every file and its image data was not possible as it was both too time-consuming and error-prone. Therefore, we implemented the following safeguards:

- The curation logs logged the number of DICOM fields after curation and were checked for consistency (expected number of tags present) and errors.
- All DICOM tags in the curated database were extracted and inspected to ensure no unwanted fields were present.
- Final sequence names were extracted to ensure that no unnecessary (and potentially identifying) images remained (e.g., Screensaves).
- Manual inspection of files, image data and DICOM metadata was conducted randomly throughout the elaboration of the curation process to identify sources of information leakage.

### Step 3: Metadata harmonisation

After running the curation script, we obtained a dataset of DICOM files with no directly or indirectly identifiable data. The script also partially harmonised the dataset by removing non-DICOM files, non-essential data and placing each file under the patient folder.

Though data collection was performed in the context of a clinical trial, which resulted in almost every patient having every sequence required, the naming of each sequence was specific to each device and institution. It was therefore necessary to harmonise the sequence names. For this step, each unique sequence name was extracted from the DICOMs and annotated by a radiologist (L.F.), in as generic a manner as possible, by including image weighting (T1, T2, DWI…), acquisition plane (sagittal, coronal or axial), anatomical location (pelvis or abdomen), whether they were 2D or 3D, and whether fat or water suppression and contrast injection were applied. In cases with equivocal names, the images were visualised by the radiologist to determine their characteristics. The resulting annotated file, as well as a simple Python reader, are available as open source at https://github.com/ThibaultSau/MRINormalizer.

This harmonisation step allowed fast, automated reading of patient sequences and information without having to parse whole folders, read DICOM metadata or create decision rules for inconsistent information. It also reduced the size of the database by removing non-essential data.

## Results

### Population

The EURAD clinical trial database included 1130 patients, among whom 947 were patients included in French centres. Among these patients, 118 were excluded because the information contained in their DICOM files could not be processed. An additional 116 patients were excluded as they did not have the minimum required imaging sequences. The final curated database contained 713 patients (Fig. 1).

Table 2 summarises the clinical findings. In the 713 patients, 897 lesions were found, including 814 adnexal lesions and 83 nonadnexal lesions. The most common adnexal lesions were epithelial tumours, representing 44% of the adnexal lesions. The prevalence of lesions in the studied population was representative of previously published populations in similar medical contexts [13, 14].

**Table 2:** Clinical data of the curated database

Population	Characteristics	No. (%)
Patients	Age (mean ± SD) (*N* = 713)	49 ± 16
	MRI findings	
	0 lesions	48 (7)
	1 lesion	462 (65)
	2 lesions	174 (24)
	3 lesions	29 (4)
	Lesion laterality (*N* = 665)^a^	
	Unilateral	514 (77)
	Multilateral	151 (23)
Lesion	Lesion size on MRI (mm) (mean ± SD)	55 ± 40
	Origin and malignancy of pelvic mass from Reference Standard (*N* = 897)	
	Adnexal masses (*N* = 814)	
	Benign	655 (80)
	Invasive	132 (16)
	Borderline	24 (3)
	Indeterminate	2 (< 1)
	Adnexal masses origin	
	Ovary	729 (90)
	Tube	47 (6)
	Tubo-ovarian	21 (3)
	Mesosalpinx	17 (2)
	Nonadnexal masses (*N* = 83)	
	Invasive	83 (100)
	Nonadnexal masses origin	
	Uterus	48 (58)
	Peritoneum	30 (36)
	Digestive tract	2 (2)
	Lymph node	1 (1)
	Nerve	1 (1)
	Urinary tract	1 (1)
	Pathology of ovarian masses (*N* = 718)^b^	
	Epithelial tumour	316 (44)
	Functional (follicle cyst)	102 (14)
	Germinal tumour	88 (12)
	Endometriosis	87 (12)
	Stromal tumour	84 (12)
	Metastasis	34 (5)
	Sex cord tumour	6 (1)
	Neuro-endocrine tumour	1 (0)

As performed in the O-RADS clinical study [15], the final diagnosis recorded for each patient was based on histology or clinical follow-up. If the lesion did not disappear or decrease at imaging follow-up, a minimum of 24 months of observation was performed (with or without imaging) from the date of the study MRI. Borderline lesions were considered malignant. In cases that underwent clinical follow-up, the origin of the pelvic mass was confirmed if there was agreement by 2 experienced readers

For all fields, percentages are reported as percentage of the total concerned population, indicated in parentheses in the subsection title. Sums of percentages may differ slightly from 100% due to rounding

^a^ Patients with no lesions were excluded

^b^ Pathology is reported for Ovarian findings excluding Adnexal torsions, Inflammatory disease, and Undifferentiated tumours

### Curation

**Fig. 4:**
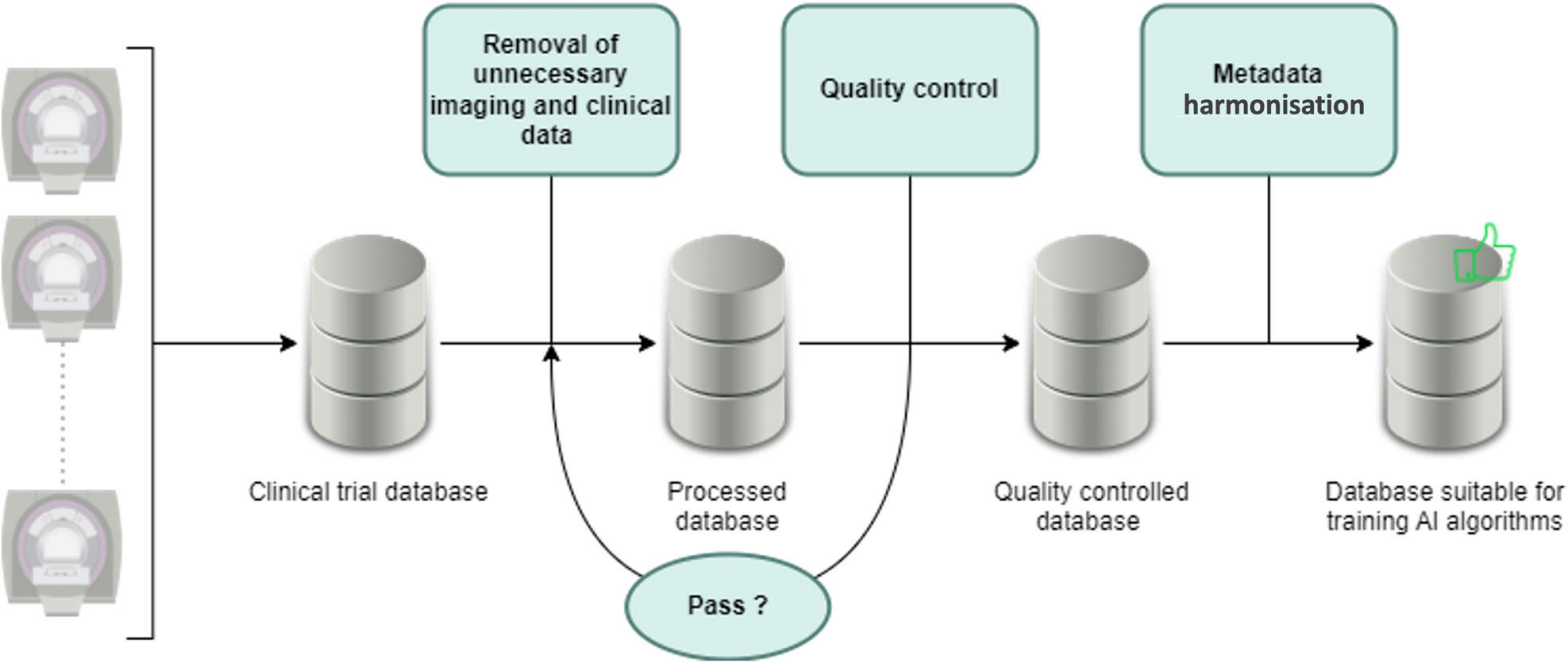
Workflow process of the software development. Unnecessary data was first removed from the original clinical trial data, images and annotations. Unnecessary data mainly were non-DICOM files, non-useful sequences for AI and non-useful DICOM metadata. A quality control process was then run to ensure that the anonymisation was carried out correctly. Quality control consisted of DICOM tag and sequence name extractions as well as reviewing the curation logs for inconsistencies and errors. All metadata were then harmonised to allow machine learning algorithms to be trained

The folder contained 2,230,922 files and 14,780 folders, representing 416 GB. The execution time of the curation script was 25 h and 12 min. After curation, the patient folder contained 713 patients, 713 folders and 1,250,308 files corresponding to 380 GB of disk space. This represented a decrease of 44% in files, 95% in folders and directories and 9% in disk space. The dataset size did not change significantly, as images represented most of the data size. However, the number of files and folders was significantly reduced, and the directory structure was simplified. Figure 4 summarises the process of creating the curation software, consisting of a loop of curation followed by automated and then manual verification, incorporating into the software and verification steps any bug found and diagnosed during the manual inspection of logs and lists of DICOM tags and sequence names.

Overall, 62 DICOM fields were considered necessary, either for the study or according to the DICOM standard for images. Table 3 lists the clinical and DICOM fields which were retained and whether and how they were transformed. This list was reviewed and approved by our institution’s Data Protection Officer (DPO), validating that the process of data impoverishment, based on the deletion and generalisation of data, allowed the dataset to be considered outside the scope of the GDPR at the current state of knowledge.

**Table 3:** List of all DICOM tags kept and the action performed on them

Field name	Field coordinates	Action taken
Image type	(‘0 × 8’, ‘0 × 8’)	Keep as-is
SOP class UID	(‘0 × 8’, ‘0 × 16’)	Replace value with a type-specific, random value
SOP instance UID	(‘0 × 8’, ‘0 × 18’)	Replace value with a type-specific, random value
Study date	(‘0 × 8’, ‘0 × 20’)	Change date to January 1st of the year
Series date	(‘0 × 8’, ‘0 × 21’)	Change date to January 1st of the year
Acquisition date	(‘0 × 8’, ‘0 × 22’)	Change date to January 1st of the year
Content date	(‘0 × 8’, ‘0 × 23’)	Change date to January 1st of the year
Study time	(‘0 × 8’, ‘0 × 30’)	Keep the field and remove its value
Content time	(‘0 × 8’, ‘0 × 33’)	Keep the field and remove its value
Accession number	(‘0 × 8’, ‘0 × 50’)	Keep the field and remove its value
Modality	(‘0 × 8’, ‘0 × 60’)	Keep as-is
Manufacturer	(‘0 × 8’, ‘0 × 70’)	Keep as-is
Referring physician’s name	(‘0 × 8’, ‘0 × 90’)	Keep the field and remove its value
Series description	(‘0 × 8’, ‘0x103e’)	Keep as-is
Manufacturer model name	(‘0 × 8’, ‘0 × 1090’)	Keep as-is
Patient name	(‘0 × 10’, ‘0 × 10’)	Change to the corresponding name in the anonymisation table
Patient ID	(‘0 × 10’, ‘0 × 20’)	Change to the corresponding name in the anonymisation table
Patient’s birth date	(‘0 × 10’, ‘0 × 30’)	Change date to January 1st, 2021
Patient’s sex	(‘0 × 10’, ‘0 × 40’)	Replace value with a type-specific, random value
Contrast/bolus agent	(‘0 × 18’, ‘0 × 10’)	Keep as-is
Scanning sequence	(‘0 × 18’, ‘0 × 20’)	Keep as-is
Sequence variant	(‘0 × 18’, ‘0 × 21’)	Keep as-is
Scan options	(‘0 × 18’, ‘0 × 22’)	Keep as-is
MR acquisition type	(‘0 × 18’, ‘0 × 23’)	Keep as-is
Sequence variant	(‘0 × 18’, ‘0 × 21’)	Keep as-is
Slice thickness	(‘0 × 18’, ‘0 × 50’)	Keep as-is
Repetition time	(‘0 × 18’, ‘0 × 80’)	Keep as-is
Echo time	(‘0 × 18’, ‘0 × 81’)	Keep as-is
Inversion time	(‘0 × 18’, ‘0 × 82’)	Keep as-is
Number of averages	(‘0 × 18’, ‘0 × 83’)	Keep as-is
Imaged nucleus	(‘0 × 18’, ‘0 × 85’)	Keep as-is
Magnetic field strength	(‘0 × 18’, ‘0 × 87’)	Keep as-is
Spacing between slices	(‘0 × 18’, ‘0 × 88’)	Keep as-is
Echo train length	(‘0 × 18’, ‘0 × 91’)	Keep as-is
Protocol name	(‘0 × 18’, ‘0 × 406’)	Replace value with a type-specific, random value
Spatial resolution	(‘0 × 18’, ‘0 × 1050’)	Keep as-is
Reconstruction diameter	(‘0 × 18’, ‘0 × 1100’)	Keep as-is
Flip angle	(‘0 × 18’, ‘0 × 1314’)	Keep as-is
Patient position	(‘0 × 18’, ‘0 × 5100’)	Keep as-is
Study instance UID	(‘0 × 20’, ‘0xd’)	Replace value with a type-specific, random value
Series instance UID	(‘0 × 20’, ‘0xe’)	Replace value with a type-specific, random value
Series number	(‘0 × 20’, ‘0 × 11’)	Keep as-is
Acquisition number	(‘0 × 20’, ‘0 × 12’)	Keep as-is
Instance number	(‘0 × 20’, ‘0 × 13’)	Keep as-is
Patient orientation	(‘0 × 20’, ‘0 × 20’)	Keep as-is
Image position (patient)	(‘0 × 20’, ‘0 × 32’)	Keep as-is
Image orientation (patient)	(‘0 × 20’, ‘0 × 37’)	Keep as-is
Images in acquisition	(‘0 × 20’, ‘0 × 1002’)	Keep as-is
Slice location	(‘0 × 20’, ‘0 × 1041’)	Keep as-is
Samples per pixel	(‘0 × 28’, ‘0 × 2’)	Keep as-is
Photometric interpretation	(‘0 × 28’, ‘0 × 4’)	Keep as-is
Rows	(‘0 × 28’, ‘0 × 10’)	Keep as-is
Columns	(‘0 × 28’, ‘0 × 11’)	Keep as-is
Pixel spacing	(‘0 × 28’, ‘0 × 30’)	Keep as-is
Pixel aspect ratio	(‘0 × 28’, ‘0 × 34’)	Keep as-is
Bits allocated	(‘0 × 28’, ‘0 × 100’)	Keep as-is
Bits stored	(‘0 × 28’, ‘0 × 101’)	Keep as-is
High bit	(‘0 × 28’, ‘0 × 102’)	Keep as-is
Pixel representation	(‘0 × 28’, ‘0 × 103’)	Keep as-is
Icon image sequence	(‘0 × 88’, ‘0 × 200’)	Keep as-is
Number of images in series	(‘0x7a1’, ‘0 × 1002’)	Keep as-is
Pixel data	(‘0x7fe0’, ‘0 × 10’)	Keep as-is

Fields not mentioned are removed by default. This list is used as the default parameters for the software we developed and is customisable in a text file

The developed curation software is available at https://github.com/ThibaultSau/SimpleDicomAnonymizer under GNU General Public License v3.0. The list of data retained, modified and suppressed in this study was used as the default settings for our curation tool, but can be modified by a user and adapted to each study or purpose.

Table 4 summarises the imaging data included in the curated dataset. The sequences mentioned are those obtained after the harmonisation step.

**Table 4:** Imaging data of the curated database

Characteristics	No. (%)
Number of sequences per patient^a^ (*n* = 713)	
1	2 (0)
2	2 (0)
3	12 (2)
4	34 (5)
5	222 (31)
6	436 (61)
7	5 (1)
Total number of sequences (*n* = 3938)	
Number of sequences in the database^b^	
T2	702 (98)
T1	689 (96)
T1FS	701 (98)
T1FSGd	589 (83)
DWI	555 (78)
ADC	10 (1)
Perf	692 (97)

*FS* fat suppression, *Gd* gadolinium, *DWI* diffusion weighted imaging, *ADC* apparent diffusion coefficient, *Perf* perfusion acquisition

Only axial or 3D sequences were retained in the dataset

^a^ Percentage is reported as percentage of the total number of patients

^b^ Percentage is reported as percentage of patients for which this sequence was acquired

## Discussion

In this study, we established a process for transforming a clinical and imaging database from a clinical trial into an exploitable base for the development of AI tools and applied it to a specific clinical trial database. Our primary objective was to develop Content-Based Image Retrieval (CBIR); however, the database is designed to support the development of segmentation, classification, or data harmonisation tools as well. To do this, we had to develop curation and harmonisation tools to ensure regulatory and technical compliance. Grouped in a package called “DATACURATOR”, we provide these tools to the community in the form of open-source, freely available Python scripts.

Our process was designed to be transposable to other clinical trial designs or imaging modalities, both in terms of the required steps and the critical issues to address. The tools shared via GitHub are similarly intended to be generalisable, allowing adaptation to each use case by modifying the configuration file. However, full standardisation is unlikely, and strict applicability to other cases may be limited. Experience shows that specificities and unforeseen issues often arise, requiring additional steps to ensure data de-identification (e.g., modalities embedding patient IDs in images or DICOM fields like acquisition parameters that must be preserved) or harmonisation (e.g., trials randomising patients across sequences that must be distinguished in naming). Decisions on which data to retain or irreversibly destroy always involve a trade-off between re-identification risk and informational value, and must be adapted to the disease, modality, and clinical context. Expertise in both medical imaging and data science is essential for this initial phase. We followed previous recommendations [15] and regulations and developed open-source tools made available to the community. However, to our knowledge, the specific steps for transforming an existing clinical trial database for secondary use in machine learning training and validation have not been previously described, despite the existence of numerous clinical trial imaging datasets. Through this restitution, we share our experience with the medical and AI communities, providing both a methodology for data curation and open-source tools to make such data computationally usable.

Although further anonymisation of data may result in loss of information, we felt it was important to do so for several reasons. The most important one is the lack of strong, universal recommendations to define when data can be considered fully and safely anonymised, either from the Health Insurance Portability and Accountability Act (HIPAA) in the USA [16], the General Data Protection Regulation (GDPR) in the EU [9], or the DICOM standard. Anonymisation ensures the highest level of patient privacy when sharing data. To date, the curated dataset is not publicly available due to remaining regulatory and storage issues.

Other curation and anonymisation tools are available [17–20], but they did not cover our use cases. Dicomanonymizer [18] is only available for MacOS, and hospital computers mainly run Microsoft Windows operating systems. The dicom-anonymizer Python package [17] and Microsoft’s Health Data Anonymisation Tools [19] are open-source programs, but did not implement all the anonymisation methods we needed. We decided to extend [17] because the software was the most specific to our use case and therefore less complex to extend. Furthermore, most of the software listed by Aryanto et al [20] required further installation steps (such as jdk or .net framework installation). We privileged the creation of standalone executable files rather than packages that required installation. We took care to make our tools expandable and to release them as open source to allow the community and users worldwide to adapt them to their own needs.

However, exploiting clinical trial databases has drawbacks and raises important questions. This approach inherits biases from the clinical trial itself (e.g., patient selection, inclusion and exclusion criteria, source population), which are typically acknowledged during the trial. Yet, identifying these biases may become impossible after information is removed from the DICOM files and clinical data. Additionally, new biases inherent to machine learning may be introduced [21]. The usefulness of models developed from such datasets depends heavily on representativity, generalisability, and trust [22]. Representativity refers to obtaining consistent results with the same hypothesis across different patient populations and methodologies. Generalisability describes a model’s ability to perform on a different patient distribution or in a real-world setting compared to the controlled development environment. Trust reflects how the end user interprets and relies on model results; while generalisability is necessary for trust, it is not sufficient. Many of these biases can be mitigated by accessing larger, high-quality datasets for training and validation, although this remains challenging for most medical AI algorithms. It is crucial to account for all these biases when building databases and designing algorithms to ensure they truly benefit both doctors and patients.

In the era of big data, we should consider whether small-scale, individual initiatives have a place or whether more global approaches should be prioritised. While initiatives such as the EHDS (Electronic Health Data Space) [23] or the CHAIMELEON [24] or EUCAIM [25] projects are being developed to facilitate large-scale access to health data in the future, we contend that there is still a need for targeted efforts to anonymise and curate existing data, as a fully universal approach for all medical data appears unrealistic. Finally, it is critical that patient well-being and privacy are prioritised throughout the database creation process and that the drive for more medical data does not overshadow these critical considerations.

## Conclusion

We developed freely accessible methods and tools embedded in an open-source package to facilitate the conversion of an existing medical database into a format suitable for training machine learning or computer vision algorithms. Our objective was to build upon the efforts deployed for a clinical trial and enable the development of AI tools based on the data and physician expertise. This work on MRI ovarian masses serves as a proof of concept, but a more systematic approach could be developed in the medical imaging community to offer guidelines and tools that would allow the successful transformation of clinical trial data into machine learning-compatible data on a large scale while remaining compliant with international regulations.

## Data Availability

A more extensive description of the data can be found reading the original paper (https://pubmed.ncbi.nlm.nih.gov/31977064/) on the O-RADS Score. A subset of the patients from this study was used in this manuscript. However, this is not a clinical article; therefore, there is no overlap with the previous publication. Data access is subject to the dataset’s specific authorisations for secondary use and may be submitted to the corresponding author.

## Abbreviations

AI: Artificial intelligence
CV: Computer vision
DICOM: Digital imaging and communications in medicine
GDPR: General Data Protection Regulation
HIPAA: Health Insurance Portability and Accountability Act
ML: Machine learning
